# Resurrection of *Bronchocela burmana* Blanford, 1878 for the Green Crested Lizard (Squamata, Agamidae) of southern Myanmar

**DOI:** 10.3897/zookeys.657.11600

**Published:** 2017-02-17

**Authors:** George R. Zug, Daniel G. Mulcahy, Jens V. Vindum

**Affiliations:** 1 Department of Vertebrate Zoology, National Museum of Natural History, Smithsonian Institution, PO Box 37012, Washington, District of Columbia, 20013-7012 USA; 2 Global Genome Initiative, National Museum of Natural History, Smithsonian Institution, PO Box 37012, Washington, District of Columbia, 20013-7012 USA; 3 Department of Herpetology, Californian Academy of Sciences, Golden Gate Park, San Francisco, California, 94103 USA

**Keywords:** Reptilia, Southeast Asia, Tanintharyi Division, Thailand, Peninsular Malaysia, morphology, molecular phylogeny, synonymy, nomenclature

## Abstract

Recent fieldwork in southern Tanintharyi revealed the presence of a small Green Crested Lizard in the wet evergreen forest. We generated mtDNA sequence data (ND2) that demonstrates that this population’s nearest relative is *Bronchocela rayaensis* Grismer et al., 2015 of Pulau Langkawi, northwestern Peninsular Malaysia and Phuket Island. Morphologically the Burmese *Bronchocela* shares many features with *Bronchocela rayaensis*, which potentially would make this recently described Thai-Malay species a synonym of *Bronchocela burmana* Blanford, 1878; however, we interpret the genetic and morphological differences to reflect evolutionary divergence and recommend the recognition of both species.

## Introduction

*Bronchocela* is a light weight among agamid lizards, rivaling *Draco* in the lightness and slenderness of its body and limbs. *Draco*’s slenderness is associated with its gliding locomotion. The slenderness and extremely long tail (~ 3× body length) of *Bronchocela* seem to be an adaptation for moving on the outer edge of the branches of shrubs and trees.

The first member of the *Bronchocela* clade to be recognized was *Agama cristatella* Kuhl, 1820 (now *Bronchocela cristatella*). This species continues to be the most commonly recognized *Bronchocela* owing to its broad distribution from Peninsular Malaysia to the Philippines and into the Lesser Sunda Islands (Grismer et al. 2015). The first mention of a *Bronchocela* in Burma (Myanmar) was in 1878 by Blanford. He recognized that a Burmese specimen differed strikingly from *Bronchocela cristatella* and the other species described to that time and named this taxon *Bronchocela burmana*. One major difference was the height, shape, and number of nuchal crest scales. Boulenger (1880) did not believe that this Burmese species was unique and synonymized *Bronchocela burmana* with *Calotes cristatellus*, a name used then because Boulenger also did not consider *Bronchocela* Kaup, 1827 to be a unique group even though *Bronchocela* had more than 50 years of recognition by other major authorities in herpetology. Malkmus (1992) appears to be the first author to use *Bronchocela* in a modern publication; however, his use was limited to *Bronchocela cristatella* presence in the Mount Kinabalu (Sabah, Borneo) area. Diong and Lim’s (1998) review of *Bronchocela cristatella* is the first formal use of *Bronchocela* with a full explanation of their decision to revive the generic name. Their decision was based on a confirmation of Scott Moody’s data in his unpublished dissertation demonstrating that *cristatella* was not closely related to other members of the *Calotes* clade.

**Figure 1.**
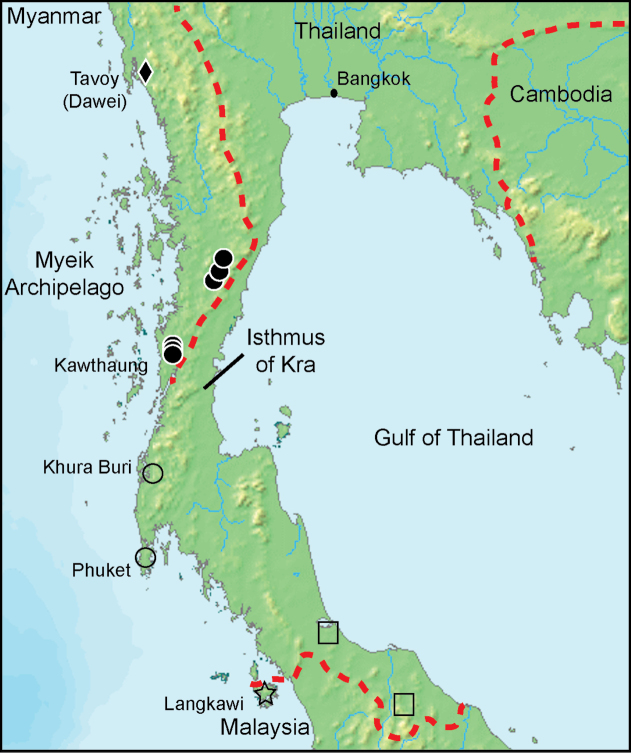
Distribution of *Bronchocela burmana* (solid circles) in southern peninsular Myanmar, Taylor’s (1963) two localities for *Bronchocela cristatella* (open squares) in southern Thailand, and *Bronchocela rayaensis* type locality (star) in northwestern Peninsular Malaysia and its newly reported localities (open circles) in Thailand (Grismer et al. 2016). A solid diamond denotes the type locality of *Bronchocela burmana*. The red dashed lines depict the political boundaries between Myanmar-Thailand, Cambodia-Thailand, and Malaysia-Thailand. Base map from: CC BY-SA 3.0, https://commons.wikimedia.org/w/index.php?curid=166887.

*Bronchocela burmana* has remained a forgotten name until the present, in part because of Boulenger’s nomenclatural authority, but also because few *Bronchocela* have been collected in Myanmar owing to its limited occurrence to southern Tanintharyi (Fig. 1) and the failure to examine closely the few specimens from southern Burma. One of us (DGM) has been participating in rapid assessment surveys of proposed national parks in southern Tanintharyi. He first discovered two specimens of *Bronchocela* in the Lenya NP area in 2015 and one in the Lenya NP Extension in 2016. We likely would have labeled these specimens as *Bronchocela cristatella* without examining them closely if part of the survey did not also include mtDNA barcoding of all collected specimens. When additional sequence data (ND2) indicated that the Lenya specimens were not closely related to *Bronchocela cristatella*, instead to the recently described *Bronchocela rayaensis* (Grismer et al., 2015) from Pulau Langkawi, northwestern Peninsular Malaysia and Phuket Island, Thailand (Grismer et al. 2016), we decided to make a morphological comparison as well.

We present below our analysis of the molecular and morphological data for the southern Burmese *Bronchocela* specimens. As our title indicates, these data demonstrate that the Burmese population is unique.

## Materials and methods

### Molecular analyses

We sequenced eight specimens of *Bronchocela*, five from the California Academy of Sciences (CAS) from the area near Kawthung, and three National Museum of Natural History-Smithsonian (USNM) specimens from the Lenya NP area (Table 1). Liver and muscle tissue samples were collected in the field during specimen preparation and preserved separately in EtOH (CAS specimens) or a DMSO/EDTA salt-saturated buffer (USNM specimens; Mulcahy et al. 2016). Specimens were subsequently fixed in 10% formalin and transferred to 70% ethanol for long-term storage at the USNM collection, housed at the National Museum of Natural History (NMNH) and at CAS. Extractions of genomic DNA were conducted on small pieces of liver or muscle tissue and run on an Auto-Genprep 965 (2011 AutoGen, Inc.), using standard phenol manufacturer protocols. Genomic DNA was eluted in 100 µl of AutoGen R9 re-suspension buffer. We sequenced the mtDNA ND2 gene because that is the locus used by Grismer et al. (2015) in their descriptions of two new species of *Bronchocela*. For our PCR reactions, we used the primers L4437–H5934 (Macey et al., 1997). Cycle-sequence reactions were performed in both directions, using the PCR primers using BigDye Terminator v3.1 Cycle Sequencing Kit’s in 0.25 × 10 µl reactions run on and ABI3730 Sequencer (2011 Life Technologies) using the 950 chemistry. Raw trace files were edited in Geneious 9.1.5 (Biomatters Ltd 2005–2016), complementary strands were aligned, edited, and inspected for translation. All sequences were deposited in GenBank under the accession numbers KY366308–KY366315. Our sequences were aligned with the ND2 samples (including the outgroups) from Grismer et al. (2015), the single new individual from Phuket Island, Thailand (Grismer et al. 2016), and another specimen in GenBank (AF128497) initially identified as *Bronchocela cristatella* (Macey et al. 2000a) and AF128495, initially identified as *Aphaniotis fusca* because these specimens were reported as being switched in GenBank (Zug et al. 2006). We used Geneious and the MUSCLE Alignment with default settings and secondarily inspected for codon alignment and translation. Grismer et al. (2016) did not deposit the ND2 sequence of their new specimen (ZMKUR0017/LSUHC12347) from Phuket Island in GenBank, but did provide us with a copy. We deposited their sequence in GenBank under the sequence number KY498356. The tRNA region of the ND2 locus required some adjustments to insure the tRNA’s were properly aligned based on secondary stem and loop structure (sensu Macey et al. 1997). The tRNA secondary structure was determined using ARWEN (Laslett and Canback 2008). We performed maximum-likelihood (ML) analyses using RAxML (v8.2.9, Stamatakis 2014) with the rapid bootstrap inferences (1000 replicates) and subsequent thorough ML search, with ND2 partitioned by codon and the tRNAs as a separate partition.

**Table 1.** Voucher information for specimens sequenced for this study. See holding institution for additional locality information.

Specimen	Locality	GenBank
USNM 587793	Myanmar: Tanintharyi, proposed Lenya National Park	KY366315
USNM 587483	Myanmar: Tanintharyi, proposed Lenya National Park	KY366313
USNM 587484	Myanmar: Tanintharyi, proposed Lenya National Park	KY366314
CAS 247755	Myanmar: Tanintharyi, Khamaukgyi Township	KY366309
CAS 247756	Myanmar: Tanintharyi, Khamaukgyi Township	KY366310
CAS 247757	Myanmar: Tanintharyi, Khamaukgyi Township	KY366311
CAS 247860	Myanmar: Tanintharyi, Khamaukgyi Township	KY366312
CAS 228481	Myanmar: Tanintharyi, Pakchan Reserve Forest	KY366308

### Morphological analyses

Hallermann began his systematic studies of *Bronchocela* in 2004 and, in a series of studies (Hallermann 2004, 2005, 2009), developed a set of characters for morphological analysis. Grismer et al. (2015) used Hallermann’s characters and introduced several new ones in his molecular and morphological study of Peninsular Malaysian populations of *Bronchocela*. Our study of morphological variation used most of the previous proposed characters and includes the following:

Measurements—snout-vent length (**SVL**), distance from the tip of snout to the vent; trunk length (**TrnkL**), distance from posterior edge of forelimb insertion to anterior edge of hindlimb insertion; tail length (**TailL**), distance from middle of vent to tip of tail; forelimb length (**ForelL**), distance (dorsal) from trunk between forelimb’s insertion to tip of third finger, not including claw (Grismer et al. 2015 listed fourth toe; we suspect that mentioned digit was incorrect); forefoot length (**ForefL**), distance from base (middle) of palm to tip of third finger, not including claw; 4^th^finger lengths (**4FingL**), distance from the juncture of the third and fourth finger to the end of the ultimate lamellae of the fourth finger, hence excluding the claw; 4^th^ toe lengths (**4ToeL**), distance from the juncture of the third and fourth toes to the end of the ultimate lamellae of fourth toe; hindfoot length (**HindfL**), distance from base (middle) of sole to tip of fourth toe, not including claw; hindlimb length (**HindlL**), distance (dorsal) from trunk between hindlimb’s insertion to tip of fourth toe, not including claw; head length (**HeadL**), distance from posterior edge of tympanum to tip of snout; head width (**HeadW**), transverse width of the head at posterior angles of jaws; head depth (**HeadD**), distance from top of head between orbits to lower surface of jaw (dentary); orbit diameter (**OrbD**), maximum horizontal distance from anterior and posterior edges of orbit (not eyeball); tympanum diameter (**TypmD**), greatest horizontal distance regardless tympanum height; body scale size dorsally (**BSC.dors**), width of parasagittal dorsal scale at midbody; body scale size ventrally (**BSC.vntl**), width of median ventral scale at midbody; nuchal crest length (**NucCrstL**), length of nuchal crest from anterior edge of anteriormost crest scale to posterior edge of posteriormost crest scale; nuchal crest height (**NucCrstH**), height of the highest/largest nuchal crest scale.

Scalation—supralabial scales (**Suplab**), number of scales between rostral scale and angle of jaw; infralabial scales (**Inflab**) numbers of scales between mental scale and angle of the jaw; loreal scales (**Loreal** = canthal scales in Hallermann [2009]), number of scales between nasal scale and granular scales of anterior border of orbit; postmental scales (**Postm**), number of throat scales (**Throat**), number of scales between end of jaws; third finger lamellae (**3ForefLm**), number of subdigital lamellae beneath third finger from first scale/lamella at digits’ insertion point to claw; fourth toe lamellae (**4HindfLm**), number of subdigital lamellae beneath fourth toe from first scale/lamella at digits’ insertion point to claw; midbody scales (**Midb**), number of scales around midbody; dorsal scales 1 (**Dorsal1**) number of dorsal scale rows with keels oriented parallel to the keels of the dorsal crest scales); dorsal scales 2 (**Dorsal2**), number of dorsal scale rows with keels oriented diagonally downward; nuchal spines (**NuchalS**), number of enlarged nuchal crest scales at least twice as large as enlarged vertebral scales; dorsal crest (**DorsCrst**), presence or absence; nuchal crest scales’ shape (**NucCrstSS**), lanceolate, cresent-shaped , or triangular.

Aspects of body shape were examined by converting paired traits to proportions, such as TailL/SVL, ForefL/ForelL, ForelL/HindlL, HeadW/HeadL. Measurements, proportions, and scale counts were analyzed by basic parametric statistics, Systat 12. We used a simple Student’s *t* test to examine dimorphism between adult females and males, significance α ≤0.05. Statistical analysis used SYSTAT 12.

Specimen number abbreviations are as follows: CAS (California Academy of Sciences), EHT (Edward H. Taylor), LSUHC (La Sierra University Herpetology Collection), USNM (United States National Museum, National Museum of Natural History), TNHC (Texas Natural History Collection, Univ. of Texas, Austin), ZMKU (Zoological Museum of Kasetsrat University, Bangkok, Thailand) and ZSI (Zoological Society of India).

## Results

**Figure 2.**
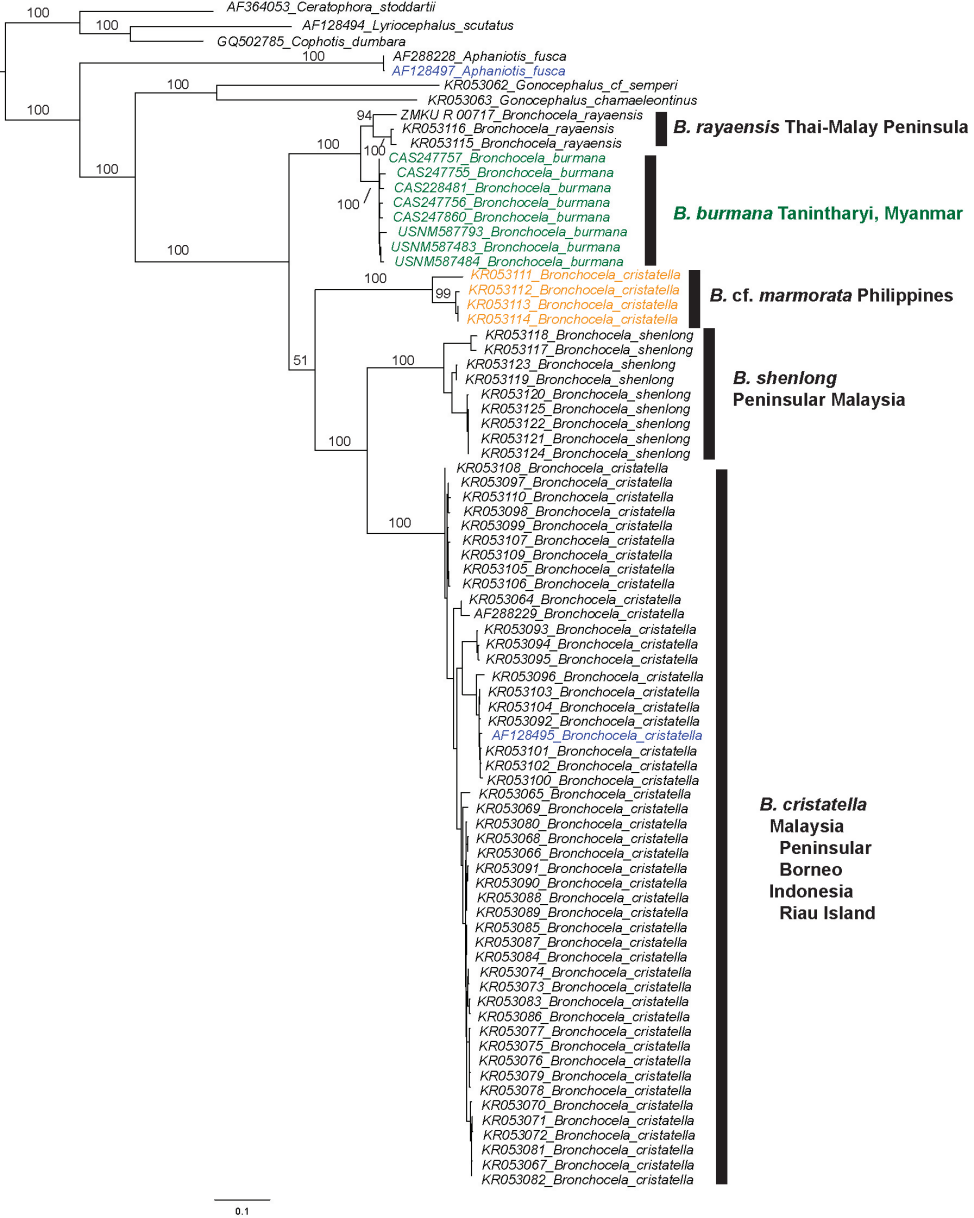
Phylogenetic relationships of *Bronchocela*. Maximum-likelihood phylogeny based on 1,416 bp of ND2 mtDNA, bootstrap values (based on 1,000 replicates) are shown for the major nodes relevant to this study. Terminals highlighted in blue were recently corrected in GenBank and the *Bronchocela cristatella* from the Philippines (in yellow) are now re-identified as Bronchocela cf. marmorata.

We obtained ND2 sequence data from eight individuals ranging in size from 1,260–1,369 bp that were 98.5–99.8% identical to one another (un-corrected sequence divergence). Our samples have a truncated origin of light strand replication (21 bp) between tRNA^ASN^ and tRNA^CYS^ that appears functional (folds using the DNA-Matthews 1999 Energy Model in Geneious) and tRNA^CYS^ shows a d-arm replacement loop (Macey et al. 1997) consistent with 45% of Draconinae agamids (Seligmann and Labra 2014). Our samples formed a well-supported clade (100%) with each other and were placed sister to *Bronchocela rayaensis* with 100% bootstrap support (Fig. 2). Our samples ranged from 93.4–95.0% identical (un-corrected sequence divergence) to the *Bronchocela rayaensis* specimens in GenBank (KR053115–KR053116, KY498356). Our analyses recovered the Phuket Island *Bronchocela rayaensis* sister to the Pulau Langkawi samples with 94% bootstrap support (Fig. 2). The rest of our tree was nearly identical to that recovered by Grismer et al. (2015) with the exception of the inclusion of *Bronchocela cristatella* (AF128495) and *Aphaniotis fusca* (AF128497); the latter was placed sister to *Aphaniotis fusca* (AF288228).

All measurements, proportions, and scalation were examined for differences between adult females (*n* = 5) and adult males (*n* =3). These samples are small so the significance of our statistical results are indicative of dimorphism but not statistical proof. Among the 33 mensural traits tested, only seven suggest dimorphism (means and ranges compared). Females average smaller (SVL 80.8, 78.8–83.8 mm) than males (SVL 88.2, 84.3–92.7 mm), fourth finger shorter in females (means, 11.0 vs. 12.6 mm 4FingL), fourth toe shorter in females (16.6 vs. 18.6 mm 4ToeL), hindlimb distinctly shorter (74.3 vs. 81.2 mm HindlL), head length less (21.2 vs. 22.6 mm HeadL), length of nuchal crest less (9.6 vs. 12.3 mm NucCrsL), and crest length to orbit diameter also shorter (150% vs. 176% NucCrsL/OrbD). No scalation features display sexual dimorphism.

To summarize the statistical results for measurements and proportions (values, mean and minimum-maximum): dimorphic traits, females’ SVL *x* = 80.8, 78.8–83.8 mm, 4FingL 11.0, 10.4–11.6 mm, 4ToeL 16.6, 15.1–17.9 mm, HindfL 29.5, 29.0–30.0 mm, HeadL 21.2, 20.3–22.0 mm, NucCrsL 9.6, 9.1–10.1 mm, NucCrsL/OrbD 150, 132–168%; males’ SVL *x* = 88.2, 84.3–92.7 mm, 4FingL 12.6, 11.3–13.9 mm, 4ToeL 18.6, 18.2–18.8 mm, HindfL 32.3, 30.9–33.1 mm; HeadL 22.6, 21.6–23.3 mm, NucCrsL 12.3, 10.6–13.3 mm, NucCrsL/OrbD 176, 166–184%; monomorphic traits (adults combined) TrunkL 42.8, 40.4–46.6 mm, TailL 276.3, 204–306 mm, ForelL 45.5, 42.2–51.5 mm, ForefL 16.3, 14.7–18.2 mm, HindlL 76.9, 70.6–86.6 mm, HindfL 30.6, 29.0–33.1 mm, HeadW 11.6, 9.5–14.1 mm, HeadH 10.2, 9.3–11.3 mm, OrbD 6.6, 5.9–7.5 mm, TympD 3.2, 2.4–3.5 mm, BSC.dors 0.6, 0.5–0.8 mm, BSC.vntl 1.7, 1.5–2.1mm, NucCrsH 1.4, 0.9–3.0, TrunkL/SVL 51, 47–56%, TailL/SVL 328, 243–361%, ForelL/SVL 54, 50–58%, HindlL/SVL 92, 86–97%, HeadL/SVL 26, 25–27%, HeadW/HeadL 54, 44–62%, HeadH/HeadL 47, 45–50%, ForefL/ForelL 35, 29–40%, HindfL/HindlL 40, 38–43%, ForelL/HindlL 21, 20–23%, 4FingL/4ToeL 67, 60–74%, TympD/OrbD 48, 40–58%, DorsS/VntlS 35, 31–39%, NucCrsL/OrbD 160, 132–184%.

The results for scalation are (values, median and minimum–maximum; adults and juvenile combined): Suplab 10, 9–11, Inflab 10, 9–11, Loreal 5, 5–6, Postm 3, 3–3, Throat 26, 25–30, 3ForefLm 28, 26–32, 4HindfLm 33, 31–38, Midbody 59, 55–67, Dorsal1 7, 6–8, Dorsal2 17, 15–21, NucCrsS 8, 6–9; nuchal spines or scales are predominantly triangular, rarely broadly crescent shaped; middorsal trunk scales are large, keeled but not elevated into a crest.

**Figure 3.**
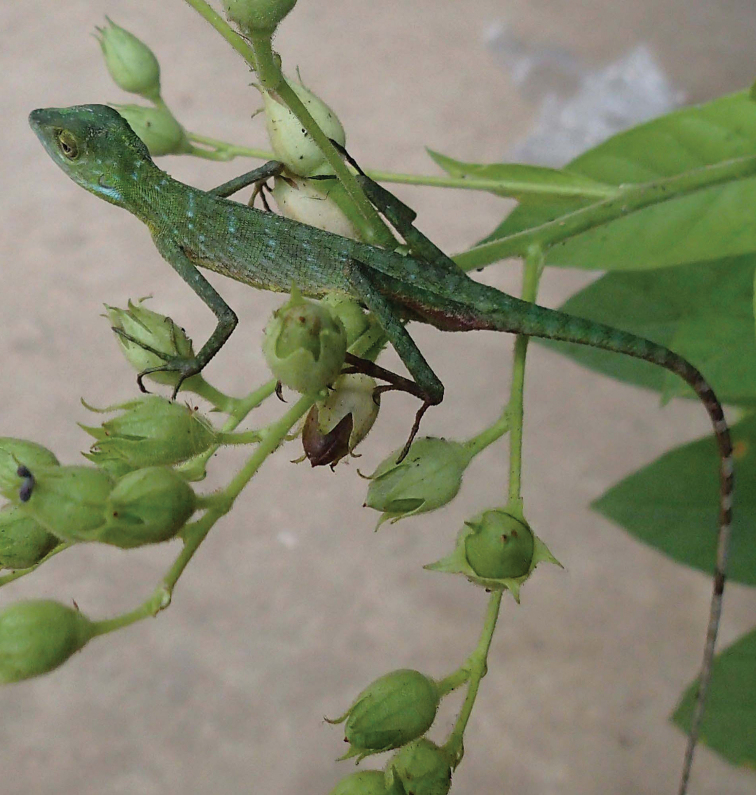
*Bronchocela burmana* Blanford, 1878 from the Lenya area (circa 11.68N 99.42E). A dorsolateral view of a living Burmese Crested Lizard, USNM 587483. Photo by DGM.

We attempted to code a few coloration traits but were unsuccessful because preservation had altered life-colors and pattern. The manner of field work and genomic tissue sampling did not permit the recording of coloration in living specimens. The general impression of the Tanintharyi *Bronchocela* is green with transverse bands of white spots on trunk and alternating bands of green and white on the tail (Fig. 3). Preserved specimens have a broadly mottled pattern of dark rufous brown and dusky turquoise dorsally and laterally. These colors form broad swatches with no consistence of position on the body although the majority (eight of nine) of the specimens have the snout and interorbital (not the superciliary area) dark. The middle third of the tail (dorsally) shows a vague banding, broad bands of medium brown and dusky tan. The venter from the tip of the chin onto the tail base is light turquoise with the chin and throat lighter that chest and abdomen. The temporal area (between orbit and tympanum) is bright rufous in eight of nine specimens although the size of this rufous patch varies from a small spot to the entire area. The eye sheath is dark, the tympanum light. The loreal area also is rufous and size of rufous patch varies from a small patch to the entire area. The ventrolateral neck folds range from uniform dark rufous to uniform turquoise. In two instances of the latter, the posterior edge of the fold bears small, ill-defined rufous spots.

For comparative purposes, we extracted the equivalent measurement and scalation data from Grismer et al.’s (2015: tables 5 & 6; 2016: table 1) description of *Bronchocela rayaensis*. Both of the types were adults, holotype a male and paratype a female, and the newly discovered Thai adult female. The following is a summary for the types (see Table 2 for summary of the three known specimens): female’s SVL 85.4 mm, HeadL 20.1 mm; male’s SVL 82.0 mm, HeadL 21.8 mm; monomorphic traits (both types) TrunkL not reported, TailL 305.5, 303–308 mm, ForelL 49.1, 48.2–50.0 mm, ForefL 15.7, 15.7–15.7 mm, HindlL 79, 78–81mm, HindfL not reported, HeadW 12.2, 12.1–12.4 mm, HeadH 10.8, 10.4–11.2 mm, OrbD not reported, TympD not reported, BSC.dors not reported, BSC.vntl not reported, NucCrsH not reported, TrunkL/SVL not reported, TailL/SVL 366, 355–376%, ForelL/SVL 59, 59–59%, HindlL/SVL 95, 91–99%, HeadL/SVL 25, 24–27%, HeadW/HeadL 58, 56–59%, HeadH/HeadL 52, 49–56%, ForefL/ForelL not reported, HindfL/HindlL 37, 37–38%, ForelL/HindlL not reported, 4FingL/4ToeL not reported, TympD/OrbD 48, 46–49%, DorsS/VntlS not reported, NucCrsL/OrbD not reported. For scalation, values are: Suplab 10, 10–11, Inflab 9, 8–10, Loreal 6, 5–7, Postm 3, 3–3, Throat not reported, 4ForefLm 32, 31–33, 4HindfLm 33 (holotype), Midbody 71 (holotype), Dorsal1 6, 5–8, Dorsal2 not reported, NucCrsS 11, 9–13; nuchal spines or scales are lanceolate; middorsal trunk scales are large, keeled but not elevated into a crest.

**Table 2.** Comparison of character metric of the three potentially allopatric species of *Bronchocela* in Myanmar, Thailand, and northern Peninsular Malaysia. Character abbreviations are defined in the Material and Methods section of the text. Numerical values are mean and range within parentheses.

**Characters**	***Bronchocela burmana*^1^**	***Bronchocela rayaensis*^2^**	***Bronchocela cristatella*^3^**
Snout–vent length, SVL (mm)	♀ 80.8 (79–84 *n=5*) ♂ 88.2 (84–93 *n=3*)	♀ 83.1–85.4 *n=2* ♂ 82.0 *n=1*	♀ to 111 ♂ 111–119
HeadW/HeadL (%)	54 (44–62)	57 (56–59)	76–94
HeadH/HeadL (%)	47 (45–50)	50 (45–56)	48–57
TympD/OrbD (%)	48 (40–54)	44 (38–49)	44–60
ForelL/SVL (%)	54 (50–58)	57 (53–59)	50–63
HindlL/SVL (%)	92 (86–97)	92 (87–99)	81–104
Nuchal crest height	low	low	high
Nuchal crest scales	8 (6–9)	10 (8–13)	8–11
Crest on trunk	No	No	Yes
Upper trunk scale row orientation: Dorsal1	7 (6–8)	6 (5–8)	6–8
Dorsal2	17 (15–21)	Not reported	17–23
4HindfLm	33 (31–38)	33 (male only)	27–34
Midbody	59 (55–67)	71 (67–74)	71–99
NucCrsS	8 (6–9)	10 (8–13)	8–10

## Discussion

The molecular (ND2) results support our Tanintharyi specimens as a discrete clade, 5–6.6% different from the closest species *Bronchocela rayaensis* available. Our samples were placed sister to *Bronchocela rayaensis*, a species known from Pulau Langkawi (island) off the west coast of Peninsular Malaysia and Phuket Island, Thailand (Fig. 2). Our phylogeny recovered the Phuket Island *Bronchocela rayaensis* sister to the Pulau Langkwai samples with 94% bootstrap support. Grismer et al. (2016) recovered it nested among the Pulau Langkwai samples, possibly because of an inaccurate alignment of the tRNA and OL region. Grismer et al. (2016) re-identified previously published *Bronchocela cristatella* samples (KR053113–KR053114; KU326254–KU326255, RMB8882–RMB8883 of Grismer et al., 2015) from Polillo Island, Philippines as *Bronchocela marmorata*, but did not update the taxonomy of those sequences in GenBank. Here, we included all samples from the Philippines from Grismer et al. (2015), the two from Polillo Island and two from Luzon Island, Philippines [KR053111–KR053112, CDS2105 (KU305472) and RMB 9878 (KU315805), respectively]. These samples form a well-supported clade and are 94.4–100% identical to one another for the ND2 sequences. Because there may be more than one lineage of *Bronchocela* in the Philippines (R. Brown, pers. com.), we refer to these specimens as Bronchocela cf. marmorata and they are sister to a clade containing *Bronchocela shenlong* from Peninsular Malaysia and *Bronchocela cristatella* from Malaysia, Borneo, and Indonesia (Pulau Natuna Besar). Though the sister relationship between *Bronchocela shenlong* and *Bronchocela cristatella* received 100% support (Fig. 2), the relationship between them and Bronchocela cf. marmorata received much less support, both in our study (51%, Fig. 2) and in that of Grismer et al. (2015, 72% ML bootstrap support); though with fewer individuals analyzed, this relationship received 100% support (Grismer et al. 2016).

We included all specimen in GenBank from Grismer et al. (2015), including outgroups, the *Bronchocela rayaensis* from Phuket Island (Grismer et al. 2016), and an additional *Bronchocela cristatella* sequence in GenBank (AF128495) and *Aphaniotis fusca* (AF128497). These specimens were reported as being switched (Zug et al., 2006), which our study confirms. The sequence AF128495 is 99% identical to *Bronchocela cristatella* from Selangor, Malaysia (KR053100–KR053102) and was placed in the *Bronchocela cristatella* clade sister to these specimens from the same state (Selangor) as TNHC57943, the voucher of AF128495 (Fig. 2). The identifications of these sequences were recently corrected in GenBank. The Fig. 5 of Macey et al. (2000b) appears to display the correct tRNA^CYS^ for these genera; however, our ARWEN model depicts the lower three base-pairs non-binding, extending the d-arm replacement loop to “AAAGTG.”

The tRNAs of Grismer et al. (2015) appear to be improperly annotated because virtually all tRNAs are annotated as transcribing in the light strand direction, whereas tRNA^ALA^, tRNA^ASN^, tRNA^CYS^, and tRNA^TYR^ transcribe on the heavy strand in all other agamids examined (e.g. Macey et al., 1997), their sequences have “tRNA^ASX^” (the transcribed product) in place of tRNA^ASN^, and none of the origin of light strand (OL) sequences are identified. Secondary structure inspection of one of their specimens (KR053067) in ARWEN reveals the tRNA^ASN^ does exists, it is transcribed in the complimentary direction, and their tRNA^ALA^ as annotated does not form a tRNA, but the bases between tRNA^TRP^ and tRNA^ASN^ do form tRNA^ALA^. Agamids, in particular, show interesting variation in the relationship between the condition of the OL and the neighboring tRNA’s d-arm replacement loop, which can be useful for the study of evolution of mtDNA replication (Seligmann and Labra 2014) and can be useful as phylogenetic information content (Macey et al. 2000b) if properly annotated.

The morphology of *Bronchocela burmana* and *Bronchocela rayaensis* are similar; however, the differences indicate adaptive divergence between the southern Burmese and Langkawi Malaysian populations. The Burmese lizards show sexual dimorphism with males larger than females with only a slight overlap. The Langkawi population seems to reverse this dimorphism with females larger than males (Table 2) although that sample includes only one adult of each sex.

The newly discovered Phuket *Bronchocela rayaensis* is a gravid female yet smaller than the paratype adult female (Table 2). As would be expected, its absolute dimensions are smaller than the typic female and surprisingly also smaller than the typic male. Also several of its body proportions (HeadH/HeadL, TympD/OrbD, ForelL/SVL, HindlL/SVL) are smaller than those of the types. Because the Phuket sample is a single individual, we note these differences, but also note that in *Bronchocela burmana* adult size remains larger, midbody and nuchal crest scales remain fewer, thereby supporting the Phuket individual as *Bronchocela rayaensis*.

*Bronchocela rayaensis* possess more nuchal crest scales than *Bronchocela burmana*—with a slight overlap 6–9 vs. 9–13. Third and fourth fingers are nearly equal in *Bronchocela rayaensis*, third finger is about half a claw length longer than fourth finger, and midbody scales 71 in holotype of *Bronchocela rayaensis* vs. 59 (55–67) scales around midbody in our Tanintharyi sample

At this time, we cannot define the distribution of *Bronchocela burmana* in Thailand. The distribution map in Chan-ard et al.’s field guide (2015: p. 94) shows a Thai occurrence of *Bronchocela cristatella* in the Tenasserim range from Khanchanburi Province southward into southern Peninsular Thailand to the Malaysia border. Their morphological description appears to mix characteristics of *Bronchocela burmana* and *Bronchocela cristatella*. We assume that this characterization demonstrates that the distribution of their Thai *Bronchocela* specimens was a mix of *Bronchocela burmana* and *Bronchocela cristatella*. The Phuket and Khura Bun specimens (Grismer et al. 2016) confirm the presence of *Bronchocela rayaensis* in Thailand, at least south of the Isthmus of Kra. In an earlier review of Thai lizards, Taylor (1963) considered *cristatella* as a *Calotes*. His description was based on a specimen from Yala Province and his illustration on an individual from Sangkhla. Both of these specimens are clearly *Bronchocela cristatella*, demonstrating that *Bronchocela cristatella* occurs in southern-most peninsular Thailand. Chan-ard et al.’s (1999) photographic atlas depicts two *Bronchocela*; the one from Malaysia (left photograph, p. 92) is an adult female *Bronchocela cristatella*, the one from Khao Soi Dao, Chanthiburi is a *Bronchocela smaragdina*, thus, of no assistance in defining the distribution of *Bronchocela burmana* in Thailand or confirming its presence there. For the present, we suggest that the northern half of the distribution of *Bronchocela cristatella* as shown in the Chan-ard et al. map (2015) is *Bronchocela burmana* from the Isthmus of Kra northward, *Bronchocela rayaensis* south of the isthmus, perhaps restricted to the western side of the Thai–Malay Peninsula, and *Bronchocela cristatella* occurs southward in the eastern lowlands into Malaysia.

*Bronchocela rayaensis* has been recently characterized by Grismer et al. 2015 and 2016 and is not repeated here. The following is a redescription of *Bronchocela burmana* based exclusively on our recently vouchered specimens from southern Tanintharyi.

### Bronchocela burmana

Taxon classificationAnimaliaSquamataAgamidae

Blanford, 1878

Burmese Green Crested Lizard

Figure 3

Bronchocela burmana Blanford, 1878, Proceedings of the Asiatic Society of Bengal 1878(6): 141.

#### Holotype.

Adult ZSI 5337 collected from ‘...near Tavoy’. We have not examined this specimen as it was not available to us.

#### Definition.

A *Bronchocela* lizard with a short nuchal crest of six to nine erect triangular crest scales; no middorsal crest of raised scales on trunk. Snout-vent length of adults range from 80 to 94 mm with tail length 240 to 360% of snout-vent length; limbs slender, forelimbs 42–52% of SVL, hindlimbs 86–97% of SVL; digits long and slender with third finger slightly longer than fourth finger, fourth toe distinctly longer that third toe; head medium sized (25–27% of SVL); head with distinct canthal ridge, narrow triangular shaped from dorsal view, length > width ≈ height and approximately 26 % of SVL; moderately large eye (OrbD/HeadL ~26–28%) and about twice diameter of tympanum (continuous with temporal surface).

#### General description.

Detailed metric and scalation features are presented above in the Results section, also Table 2. *Bronchocela burmana* is a slender green lizard with long tail, usually 2.5–3.5X snout-vent length. In spite of its 80 to 94 mm body length, its slenderness and thin legs give it a delicate appearance and make it immediately recognizable among the other lizards of southern Tanintharyi.

In life, *Bronchocela burmana* appears uniformly green (Fig. 2). Preservation changed the overall coloration to light olive but highlights a light rufous vertical bar in the temporal area.

#### Distribution.

*Bronchocela burmana* is presently confirmed for only southern Tanintharyi Division, Myanmar (Fig. 1). The type-locality is given as Tavoy, now Dawei; however, two years of surveys in both the dry and wet seasons at the Tanintharyi Nature Reserve, just north of Dawei, did not confirm the presence of this species in the former Tavoy area (approximately 350 km N of Lenya). The morphological description of *Bronchocela cristatella* in Chan-ard et al.’s field guide (2015: p. 94) appears to be a composite of *Bronchocela burmana* and *Bronchocela cristatella*. As noted in the preceding section, we interpret their distribution map also to be a composite of the two species’ occurrence in western Thailand. We suggest that the two species are allopatric with *Bronchocela burmana* in the north, likely north of the Isthmus of Kra and *Bronchocela cristatella* in the south. Taylor’s description and photograph of *Bronchocela cristatella* (1963) are definitely of that species and are plotted (Fig. 1) to confirm its occurrence in southern-most Thailand.

#### Etymology.

Blanford did not explain his choice of *burmana*. His selection seems obvious as it was the first *Bronchocela* from British Burma and distinctly different from *Bronchocela cristatella* and the other species recognized at that time.

## Supplementary Material

XML Treatment for Bronchocela burmana

## Specimens examined

*Bronchocela burmana*.

Myanmar; Tanintharyi Division: CAS247755 – CAS247757, CAS24786, USNM587775 – USNM587776, USNM587483 – USNM587484, USNM587793.

*Bronchocela cristatella*.

Malaysia; Johore Province: USNM29576, USNM29579; Selangor Province USNM129482, USNM141704 – USNM141706.

Thailand; Songgkhla Province: EHT 349; Yala Province EHT 134; data from Taylor 1963.

*Bronchocela rayaensis*.

Malaysia; Kedah Province: data from Grismer et al. 2015 and 2016.
